# PReS-FINAL-1013: Magnetic resonance imaging as a non-invasive tool to assess muscle edema in juvenile dermatomyositis. Return to normal after treatment

**DOI:** 10.1186/1546-0096-11-S2-P10

**Published:** 2013-12-05

**Authors:** E Iglesias, M Navallas, L Riaza, V Torrente-Segarra, R Bou, S Ricart, MI González, J Sánchez, J Antón

**Affiliations:** 1 Unit of Pediatric Rheumatology, Department of Pediatrics, Barcelona, Spain; 2 Department of Radiology, Hospital Saint Joan de Déu, Esplugues de Llobregat, Barcelona, Spain

## Introduction

Juvenile Dermatomyositis (JDM) is the most common idiopathic inflammatory myopathy in childhood, a systemic vasculopathy that usually affects skin and skeletal muscle but also can affect gastrointestinal tract and other organs. Diagnosis is based on Bohan and Peter's criteria and the goal of treatment includes control of skin and muscle symptoms and prevent disease complications. Stepwise aggressive treatment decreases JDM activity and improves long-term outcome.

Role of magnetic resonance imaging (MRI) as a non invasive tool to asses muscle edema have increased last years with a diagnostic sensitivity being 76%, compared with 64% for creatine kinase and ability to distinguish between active JDM patients and inactive healthy children. Attempt to establish MRI scores have been done whether evaluating thigh muscle groups or whole body MRI.

## Objectives

To evaluate muscle edema on arm and tight MRI of our JDM patients after and before treatment.

## Methods

We made a descriptive retrospective review of our JDM patients with muscle MRI before and after treatment. Muscle edema was assessed independently by two radiologists on four arm and thigh muscles of both sides (deltoid, supraspinatus, infraspinatus and subscapular and gluteals, hamstrings, quadriceps and adductors) on a 4-point-score (none = 0, mild = 1, moderade = 2, severe = 3) before and after treatment. We also scored the presence of fascia and soft-tissue edema (absent = 0, present = 1).

## Results

8 patients were included. All have pathological MRI before treatment. We did not find differences between each side. The mean scores for each muscle, arm and tight before and after treatment are given in table 1 and 2 respectively. We did not find differences between arm and tight muscle edema if we compared whole means but individually supraspinatus, infraspinatus and gluteals were the most affected muscles. Almost all muscle return to normal signal on MRI after treatment and the ones which still remained slightly pathological were the most affected before treatment. Fascia and soft tissue edema disappeared after treatment.

## Conclusion

MRI is an important not invasive tool to evaluate muscle edema in JDM patients. Our results, as others have suggested in the past, shown that tight MRI could be enough for studying JDM patients at diagnosis. Whole body MRI seems promising not only for diagnose but also for follow up and as an outcome tool in these patients.

## Disclosure of interest

None declared.

